# Functional responses of lymphoid stromal cells to Toll-like receptor stimuli *in vitro*

**DOI:** 10.1093/discim/kyag008

**Published:** 2026-04-21

**Authors:** Isabella Cinti, Kassandra Vezyrgianni, Alice E Denton

**Affiliations:** Department of Immunology and Inflammation, Imperial College London, London, UK; Department of Immunology and Inflammation, Imperial College London, London, UK; Department of Immunology and Inflammation, Imperial College London, London, UK

**Keywords:** lymphoid stromal cells, Toll-like receptors, inflammation

## Abstract

**Introduction:**

Lymphoid stromal cells control the spatial organization of lymphoid tissues and respond dynamically during immune reactions, undergoing transcriptional and phenotypic changes that can influence immune responses. The exogenous signals to which lymphoid stromal cells respond, and how, are still being defined.

**Methods:**

Lymphoid stromal cells express Toll-like receptors (TLRs), but it has been difficult to separate the role of the stromal cell response to TLR ligands from that of immune cells. To understand this better, we established a method to culture primary lymphoid stromal cells to study their behaviour *in vitro*.

**Results:**

We found that co-culturing fibroblasts with lymphatic endothelial cells produced the most stable cultures, in contrast to pure fibroblast cultures. Lymphoid stromal cells directly sensed TLR ligands, upregulating the cell adhesion molecules intercellular adhesion molecule-1 and vascular cell adhesion molecule-1 in response to TLR3 and TLR4 stimulation. Lymphoid stromal cells also secreted cytokines and chemokines in response to TLR4 ligation.

**Conclusion:**

Lymphoid stromal cells can respond directly to pathogen- and damage-associated molecules, potentially modulating the immunological outcome.

## Graphical Abstract

**Graphical abstract:**
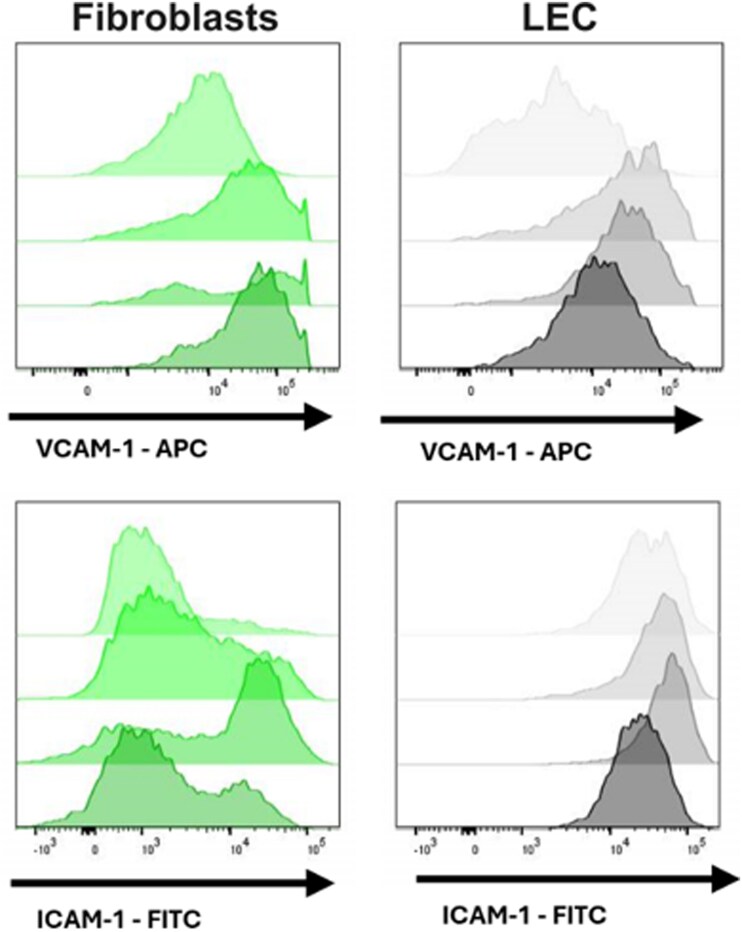

## Introduction

The lymph node is an important site of immunological activity where leukocytes converge to develop an immune response during infection or immunization. The lymph node is compartmentalized into distinct zones that house specific immune cells and coordinate their responses, and these regions are governed by different stromal cell types, e.g. lymphatic endothelial cells (LECs) and lymphoid fibroblasts. LECs surround and infiltrate the lymph node, facilitating the movement of immune cells from the lymphatics into the cortex and paracortex of the lymph node. They also form lymphatic sinuses, which support the exit of lymphocytes [1]. Once in the lymph node, leukocyte movement, localization, and behaviour are coordinated by lymphoid fibroblasts. Lymphoid fibroblasts act as a tissue scaffold and provide survival factors, adhesion molecules, and chemotactic cues for correct immune cell interaction and localization in the lymph node [2–5]. The fibroblast network dictates the spatial organization of immune cells across the lymph node, and the disruption of such organization impairs the generation of adaptive immunity [6]. Similar disruption is seen in aged tissues and is associated with poor T:B cell segregation and reduced germinal centre responses [7–11].

Despite making up a small fraction of cells in lymph nodes, fibroblasts provide structural integrity to the whole organ and instruct its compartmentalization [12]. Lymphoid fibroblasts also respond to immune perturbation *in vivo* in mouse models, sensing inflammation and upregulating cytokines and chemokines [13] and remodelling lymph node architecture [14, 15]. A simple measure of fibroblast activation is the upregulation of the adhesion molecules intercellular adhesion molecule-1 (ICAM-1) and vascular cell adhesion molecule-1 (VCAM-1) [15] and the glycoprotein podoplanin (Pdpn) [11], an important cellular interaction between fibroblasts and dendritic cells as defined in mouse models [16–18]. The direct signals that mediate lymphoid fibroblast responses are not, however, clearly defined. We have previously published that the addition of TLR4 ligands to vaccination in mice enhances lymphoid fibroblast activation [11], suggesting that lymphoid fibroblasts can directly respond to inflammatory stimuli. In this study, we established methods to culture mouse lymphoid fibroblasts and LECs, finding that lymphoid fibroblast maintenance was compromised in the absence of LECs. This was unique to lymphoid fibroblasts, as lung fibroblasts readily expanded as a pure culture. We then tested lymphoid stromal cell responses to TLR stimulation. Both lymphoid fibroblasts and LECs responded to TLR4 ligands *in vitro*, upregulating ICAM-1 and VCAM-1, but not Pdpn, and producing inflammatory cytokines. We found similar responses to TLR3 stimulation, where ICAM-1 and VCAM-1, but not Pdpn, were induced in both lymphoid fibroblasts and LECs, suggesting a path for direct sensing of pathogen-derived signals by lymphoid stromal cells that may impact immune cell behaviour and function.

## Materials and methods

### Animal husbandry

All animal experiments were carried out in accordance with the UK Animals Scientific Procedures Act 1986 and were approved by Imperial College London’s Animal Welfare and Ethical Review Body and the UK Home Office. Wild-type C57BL/6 mice were purchased from Charles River; *Tlr4*^−/−^ [19] and *Myd88*^−/−^ [20] mice were bred and housed in specific pathogen-free conditions at Imperial College London’s Central Biomedical Services, maintained in individually ventilated cages with free access to food and water.

### Stromal cell digestion

Peripheral lymph nodes (axillary, brachial, and inguinal) from 2–3 mice were harvested, pooled, and digested enzymatically as described [21]. Briefly, lymph nodes were cleared of adipose tissue, pierced with fine forceps, and placed in RMPI containing 0.1 mg/ml DNase I (Roche, cat # 10104159001), 0.8 mg/ml Dispase II (Roche, cat # 4942078001), and 0.2 mg/ml Collagenase P (Roche, cat # 11213865001) at 37°C. Tubes were inverted at 5-min intervals; at 15 min, the lymph nodes were triturated, contents allowed to settle, and the digestion buffer containing released cells was collected in a 15-ml tube containing 5 ml of fibroblast culture media: DMEM with GlutaMAX (Thermo Fisher, cat # 31966047) containing 10% fetal bovine serum (FBS) (PANBIOTECH), 1% penicillin/streptomycin (Sigma), and 1% insulin–transferrin–selenium (ITS) (Thermo Fisher, cat # 41400045). Fresh digestion buffer was added, and the samples were triturated every 5 min, with supernatant containing released cells collected after 15 min. This was repeated a third time. Cell suspensions were centrifuged at 200 × *g* for 10 min and resuspended in warm fibroblast media. Cell suspensions were passed through a 100-μm sieve, seeded in a T75-cm^2^ culture flask, and incubated at 37°C, 5% CO_2_. The following day, non-adherent immune cells were removed by gently washing the flask surface with warm fibroblast media. Media was not refreshed until cells were passaged 4 days after seeding. For lung digestions, lungs were dissected, minced with scissors, then digested in 1 ml of digestion buffer, as described for lymph node isolation, with the addition of a brief 10-s pulse in the microfuge to encourage air-containing fragments to sink prior to collecting the supernatant.

### Stromal cell passage and culture

Cells were expanded to 175 cm^2^ for 5 (for magnetic enrichment) or 7 (unenriched) days. Cultures were grown for 14 days, and up to 21 days, for experiments. Primary lymphoid fibroblasts were passaged three times a week, when they reached 80% confluence; media was not refreshed between passages. Adherent cells were rinsed with PBS and detached with 5 ml Accutase (BioLegend, cat # 423201) for 10 min at 37°C, 5% CO_2_. Accutase was neutralized with 10 ml of warm fibroblast media. Cells were centrifuged at 200 × *g* for 10 min and re-seeded in new flasks at a 1:5 ratio or counted for use in *in vitro* assays. For lung fibroblast cultures, only the platelet-derived growth factor receptor alpha (PDGFRα)-enriched cells were maintained in culture. Cells were harvested and subcultured every 7 days for a maximum of 28 days, using the same procedure as for lymphoid fibroblasts.

### MACS purification of fibroblasts by CD31 depletion or PDGFRα enrichment

Cell suspensions were depleted of CD31^+^ cells according to the positive selection protocol with LS columns by Miltenyi Biotech. Briefly, cultured primary lymphoid fibroblasts were harvested at 5 days, counted, and resuspended in 500 µl PBS/2 mM EDTA; 1.5 µg of CD31-biotin (clone MEC13.3, BioLegend, cat # 102503) or anti-PDGFRα (clone APA5, BioLegend, cat # 135910) was added per 1 × 10^7^ cells and incubated for 15 min at 4°C. Cells were washed with PBS/2 mM EDTA and centrifuged at 200 × *g* for 10 min; 7–10 µl of streptavidin microbeads (Miltenyi Biotech, cat # 130-048-101) was added per 1 × 10^7^ cells and incubated for 15 min at 4°C. Cells were washed and centrifuged as above and resuspended in 500 µl PBS/2 mM EDTA/0.5% BSA and passed over LS columns per manufacturer’s instructions. The flow-through containing unlabelled cells and the column-bound labelled cells were both collected and subcultured as described above. For PDGFRα enrichment of lung fibroblasts, the same procedure was followed using between 0.25 and 0.35 µg of anti-PDGFRα biotinylated antibody per 1 × 10^7^ cells.

### Purification of lymphoid fibroblasts by cell sorting

Lymph node suspensions obtained following fibroblast digestion were depleted of CD45^+^ cells using CD45 MicroBeads (Miltenyi Biotech, cat # 130-052-301) as previously described [11, 21], according to the manufacturer’s protocol. CD45^−^ cells were centrifuged at 200 × *g* for 10 min, resuspended in 50 µl BD Brilliant Stain Buffer, and stained with antibodies directed against CD45, CD31, and Pdpn (Table 1) for 45 min at 4°C. Samples were washed, centrifuged, and resuspended in PBS/2% FBS. Enriched CD45^−^ cells were further purified by cell sorting for Pdpn^+^ CD31^−^ and Pdpn^+^ CD31^+^ cells using an AriaFusion (BD Biosciences); 2 × 10^5^ Pdpn^+^ CD31^−^ fibroblasts were collected in PBS/10% FBS, then resuspended in warm fibroblast culture media and seeded in T75-cm^2^ flasks.

**Table 1.** Antibodies used for flow cytometry and cell sorting of lymphoid fibroblasts.

Fluorophore	Marker	Clone	Cat #, manufacturer	Dilution factor
FITC	ICAM-1 (CD54)	YN1/1.7.4	116106, BioLegend	1000
PerCP-Cy5.5	CD31	MEC13.31	102522, BioLegend	1000
APC	VCAM-1 (CD106)	429 (MVCAM.A)	105712, BioLegend	1000
E780	Fixable viability dye		65-0865-18, Thermo Fisher	1000 or 2000
PE	TLR4/MD2	MTS510	12-9924-81, Thermo Fisher	400
PE-Cy7	Podoplanin	8.1.1	25-5381-82, Thermo Fisher	1000
BV421	Podoplanin	8.1.1	127423, BioLegend	600 or 1000
Pacific Blue	ICAM-1	YN1/1.7.4	116116, BioLegend	1000
BV510	CD45	30-F11	103138, BioLegend	2000
BV605	Streptavidin		405229, BioLegend	1000
BV711	Streptavidin		405241, BioLegend	1000
BV785	CD45	30-F11	103149, BioLegend	300
BV785	CD31	390	102435, BioLegend	2000
Biotinylated	PDGFRα (CD140a)	APA5	135910, BioLegend	

### TLR4 internalization assay

Cultured primary lymphoid fibroblasts were harvested and seeded on Day 0 at 1 × 10^5^ cells/ml in a 12-well plate in 1 ml fibroblast culture media without ITS. The following day, they were stimulated with 1 µg/ml of lipopolysaccharide (LPS) (0127: B4). Cells were harvested at 5-, 10-, 30-, 60-, and 90-min post-stimulation with 1 ml Accutase. Cells were stained for flow cytometry using antibodies directed against TLR4, Pdpn, and CD31 (Table 1) at 4°C for 1 h in PBS/2 mM EDTA/2%FBS, fixed at 4°C for 30 min with the Foxp3/Transcription Factor Staining Buffer kit (Thermo Fisher, cat # 00-5523-00). Samples were washed in 1 ml 1× Perm/Wash buffer, pelleted, and resuspended in PBS/2 mM EDTA/2%FBS for data collection. Data were acquired on a BD LSR Fortessa X-20 using FACSDiva Software. TLR4 internalization was determined by gating for TLR4^+^ cells after stimulation, expressed relative to staining on unstimulated cells; TLR4 expression was determined by staining on cultured *Tlr4*^−/−^ lymphoid stromal cells.

### Flow cytometric phenotyping

Cultured primary lymphoid fibroblasts were harvested and seeded on Day 0 at 1 × 10^5^ cells/ml in a 12-well plate in 1 ml fibroblast culture media without ITS. The following day, they were stimulated with 1 µg/ml of LPS (0127: B4) and cells harvested 6, 24, and 48 h later with 1 ml Accutase. Cells were stained for flow cytometry using antibodies directed against VCAM, ICAM, Pdpn, and CD31 (Table 1) at 4°C for 1 h in PBS/2 mM EDTA/2%FBS, fixed at 4°C for 30 min with the Foxp3/Transcription Factor Staining Buffer kit. Samples were washed in 1 ml 1× Perm/Wash buffer, pelleted, and resuspended in PBS/2 mM EDTA/2%FBS for data collection. Data were acquired on a BD LSR Fortessa X-20 using FACSDiva Software.

### RNA extraction and qRT-PCR

RNA extraction with the RNAeasy plus mini kit (Qiagen, cat # 74134) was carried out according to the manufacturer’s instructions. Briefly, lymphoid primary fibroblasts were harvested and seeded on Day 0 at 2 × 10^6^ cells/ml in a 100-mm Petri dish in 10 ml fibroblast culture media without ITS. The following day, fibroblasts were stimulated with 1 µg/ml of LPS (0127: B4), and cells were harvested 24 and 48 h later with a cell scraper. Cells were collected in PBS and placed immediately on ice, centrifuged at 450 × *g* for 5 min at 4°C, and placed back on ice. Cell pellets were lysed with 350 µl of RLT buffer by vortexing for 30 s, and RNA was extracted per the manufacturer’s instructions, including the genomic DNA elimination step. RNA was eluted in 20 µl of RNAse-free water, quantified by Nanodrop, and stored at −70°C. cDNA was synthesized with the iSCRIPT cDNA synthesis kit (Bio-Rad, cat # 1708891) according to the manufacturer’s protocol, including no reverse transcripase controls. qRT-PCR was conducted with TaqMan primer-probes (Table 2), per manufacturer’s instructions with TaqMan Fast Advanced Master Mix (Thermo Fisher, cat # 4444557) in 384-well plates with 50 ng cDNA template in a 10-μL reaction. qPCR was performed on Fast Run mode in the ViiA 7 Real-Time PCR System (Bio-Rad).

**Table 2.** Primer probes used for mRNA expression analyses.

Gene	Probe
*Il6*	Mm00446190_m1
*Saa3*	Mm00441203_m1
*Tnf*	Mm00443258_m1
*Ccl5*	Mm01302427_m1
*Cxcl9*	Mm00434946_m1
*Irf7*	Mm00516791_g1

### LEGENDplex assay

Supernatants were collected 6, 24, and 48 h after LPS stimulation for surface marker detection, pelleted to remove contaminating cells, aliquoted, and stored at −20°C. The levels of cytokines were detected using LEGENDplex anti-viral panel using 25 μL of supernatant as per manufacturer’s instructions for the mouse anti-virus response panel (BioLegend, cat # 740621). Data were acquired on a BD LSRFortessa X-20 and analysed according to the manufacturer’s instructions.

### Statistical analyses

Normalcy was not assumed nor tested owing to the lower number of biological replicates (*n* = 3–10). For two-group analyses, a Mann–Whitney test was used. For three-group analyses, a two-way ANOVA was used with appropriate *post hoc* corrections as outlined in the figure legend. Statistics were performed with Prism 10 (GraphPad). A *P-*value of <0.05 was considered statistically significant, where *P < 0.05, **P < 0.01, ***P < 0.001, and ****P < 0.0001. The number of biological replicates is stated in the figure legend.

## Results

### Primary lymphoid stromal cultures are composed of fibroblasts and lymphatic endothelial cells

**Figure 1:**
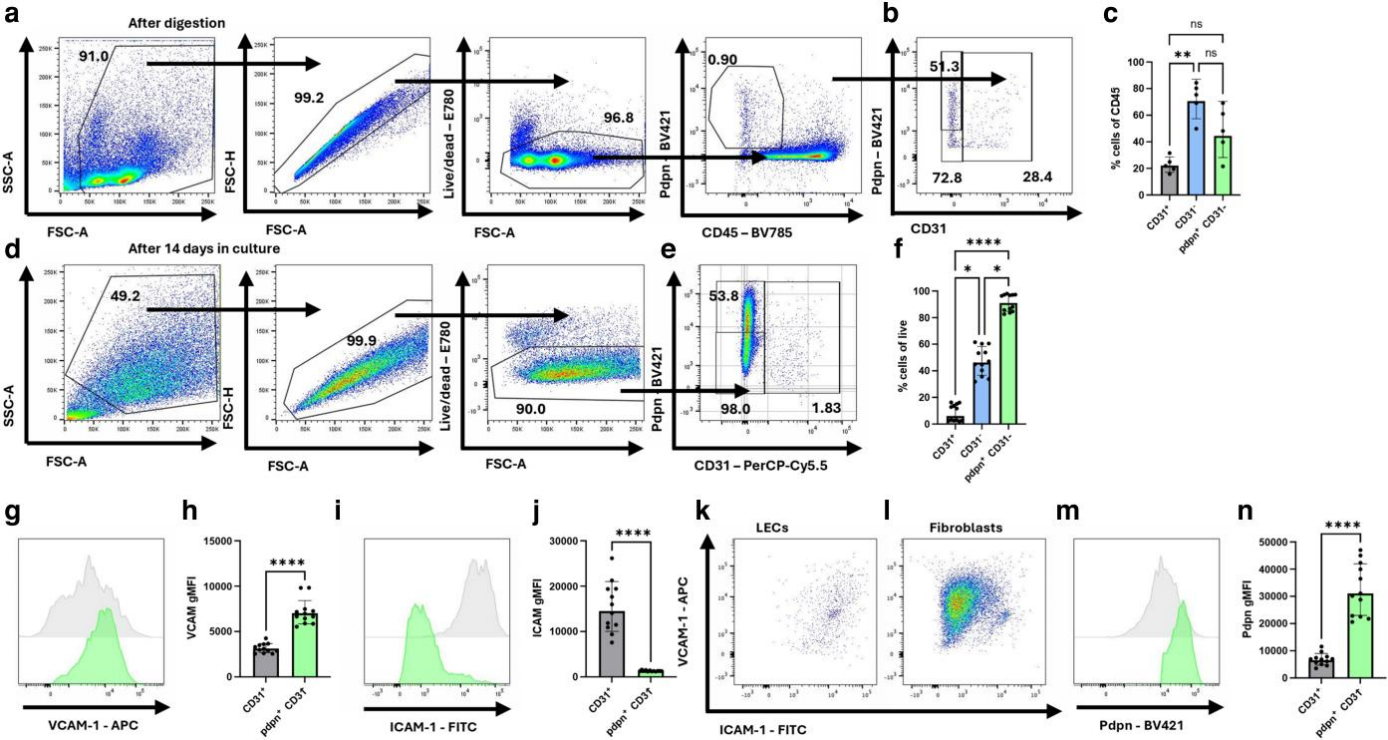
Primary lymphoid stromal cell cultures comprise mixed cell populations. Lymphoid stromal cells were obtained from peripheral lymph nodes. (a–c) Analysis of freshly digested lymphoid stromal cells. (a) Representative gating strategy for analysis of *ex vivo* lymphoid stromal cells, gating for cells, single cells, live cells, Pdpn^+^ CD45^−^ cells (non-immune cells). (b) Representative flow profile (b) and the proportion (c) of cells gated on Pdpn^+^ CD45^−^ cells: CD31^+^ (EC), CD31^−^ (putative pericytes and fibroblasts), and Pdpn^+^ CD31^−^ (only fibroblasts) cells. (d–l) Analysis of cultured primary lymphoid stromal cells. Representative gating strategy (d), gating for cells, single cells, live cells. Representative flow profile (e) and proportion (f) of cells gated on live cells: CD31^+^ (EC), CD31^−^ (putative pericytes and fibroblasts), and Pdpn^+^ CD31^−^ (fibroblasts) cells. Cell surface phenotype of cells in culture was determined by measuring the gMFI of antibody staining for VCAM-1 (g, h), ICAM-1 (i, j) in both fibroblasts and LECs. Representative flow plots show VCAM-1 and ICAM-1 staining of LECs (k) and fibroblasts (l). gMFI of antibody staining for Pdpn (m, n) in both fibroblasts and LECs is shown. Each data point represents a culture from an individual mouse, and each data point is considered independent. Each symbol represents a biological replicate, and lines indicate the geometric mean. Statistical significance was determined using a Kruskal–Wallis test with Dunn’s multiple comparison test (c, f), and Mann–Whitney test (h, j, n). *P < 0.05, **P < 0.01, and ****P < 0.0001.

To establish a protocol for the culture of primary lymphoid stromal cells, peripheral lymph nodes were enzymatically digested following the protocol described by Fletcher *et al*. [21] and expanded *in vitro* for 14 days. Cells collected immediately after digestion (Fig. 1a–c) and after 14 days in culture (Fig. 1d–f) were assessed for the expression of endothelial and fibroblast markers by flow cytometry, gating for CD31^+^ ECs, CD31^−^ stromal cells, and Pdpn^+^ CD31^−^ lymphoid fibroblasts. Freshly digested cells and cultured cells were gated differently: while freshly digested fibroblasts and endothelial cells were gated on CD45^−^Pdpn^+^ cells (Fig. 1a–c), cultured fibroblasts and ECs were gated directly on live cells (Fig. 1d–f). After digestion, <1% of the cells were CD45^−^ stromal cells, which comprised a mixed population of cells that was 20% ECs and 40–60% fibroblasts (Fig. 1a–c). ECs were a mixture of Pdpn^+^ LECs and Pdpn^−^ blood endothelial cells, and fibroblasts were equally split between Pdpn^+^ and Pdpn^−^ subsets (Fig. 1b and c). We next tested the phenotype of fibroblasts after 14 days in culture, examining purity and the expression of adhesion molecules involved in lymphocyte–fibroblast interactions, ICAM-1 and VCAM-1. LECs and fibroblasts had adapted to culture, resulting in differences in Pdpn and CD31 expression (Fig. 1e), and exhibited distinct patterns of adhesion molecule expression: both populations expressed VCAM-1, with fibroblasts having a higher geometric mean fluorescence intensity (gMFI) of staining than LECs (Fig. 1g and h); cultured fibroblasts had lost ICAM-1, while cultured LECs maintained ICAM-1 expression (Fig. 1i and j). Expression of VCAM-1 typically correlated with ICAM-1 in unstimulated LECs (Fig. 1k) and fibroblasts (Fig. 1l). In both LECs and fibroblasts, higher ICAM-1 expression tended to correlate with increased VCAM-1 expression (Fig. 1k and l). Finally, Pdpn^+^ fibroblasts maintained higher expression of this molecule for the duration of the culture period (Fig. 1m and n).

### Mixed LEC–fibroblast cultures promote culture longevity

**Figure 2:**
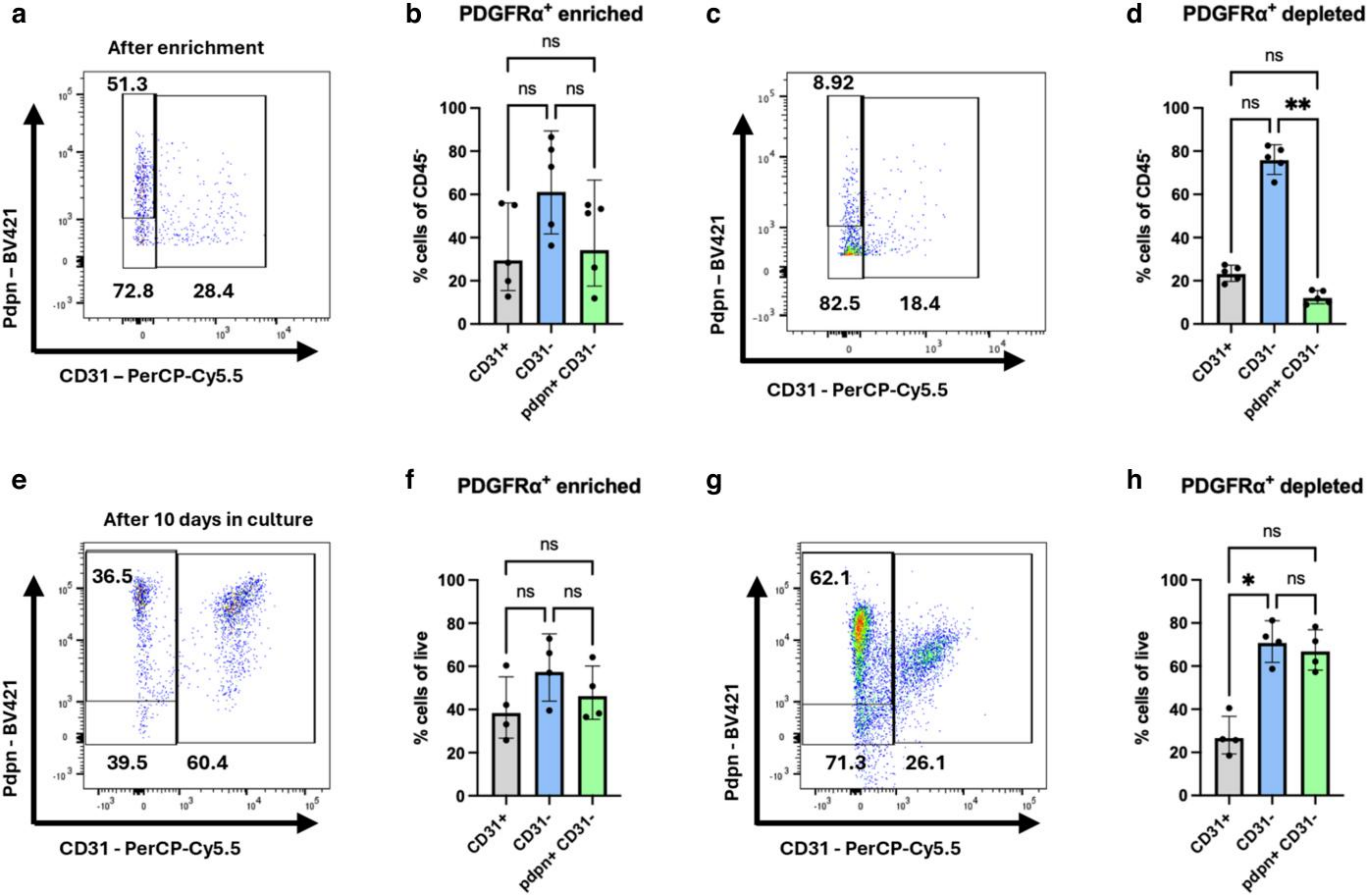
PDGFRα^+^ enrichment does not lead to pure fibroblast cultures. Lymphoid stromal cells were obtained from peripheral lymph nodes, enriched based on PDGFRα-binding, cultured for 10 days, and the LEC and fibroblast composition was determined by flow cytometry. (a–d) Staining directly after enrichment, showing PDGFRα^+^-enriched (a, b) and -depleted (c, d) samples. (e–h) Staining after 10 days in culture, showing PDGFRα^+^-enriched (e, f) and -depleted (g, h) samples. In (b, d, f, h), each data point represents a culture from an individual mouse, and each data point is considered independent. Each symbol represents a biological replicate, and lines indicate the geometric mean. Statistical significance was determined using a Kruskal–Wallis test with Dunn’s multiple comparison test (b, d, f, h). *P < 0.05 and **P < 0.01.

To obtain a culture of pure lymphoid fibroblasts, two protocols to enrich for Pdpn^+^ CD31^−^ cells were tested, enriching for PDGFRα^+^ cells or depleting CD31^+^ cells. We elected to enrich using PDGFRα as this protein is expressed on most fibroblasts, while Pdpn is also expressed on LECs [1] and inflammatory macrophages [22]. PDGFRα^+^ cell enrichment was carried out immediately after enzymatic digestion and both the PDGFRα^+^-enriched and -depleted (‘flow-through’) cells were expanded *in vitro* for 10 days. PDGFRα^+^-based enrichment did not increase the proportion of Pdpn^+^ CD31^−^ cells (amongst CD45^−^ cells) prior to culture (Fig. 1c, 2a and b); we further observed depletion of both LECs (CD31^+^) and fibroblasts (Pdpn^+^ CD31^−^) in the PDGFRα^+^-depleted sample (Fig. 2c and d). After *in vitro* expansion for 10 days, most PDGFRα^+^-enriched samples had poor purity. Up to 60% of the cells obtained 10 days after culture of PDGFRα^+^-enriched cells were CD31^+^ cells (Fig. 2e and f). In contrast, the unlabelled fraction (‘PDGFRα^+^-depleted’) still contained a substantial proportion of fibroblasts (60%) after 10 days in culture (Fig. 2g and h). Therefore, this enrichment protocol did not offer a solution to obtain pure lymphoid fibroblast cultures.

**Figure 3:**
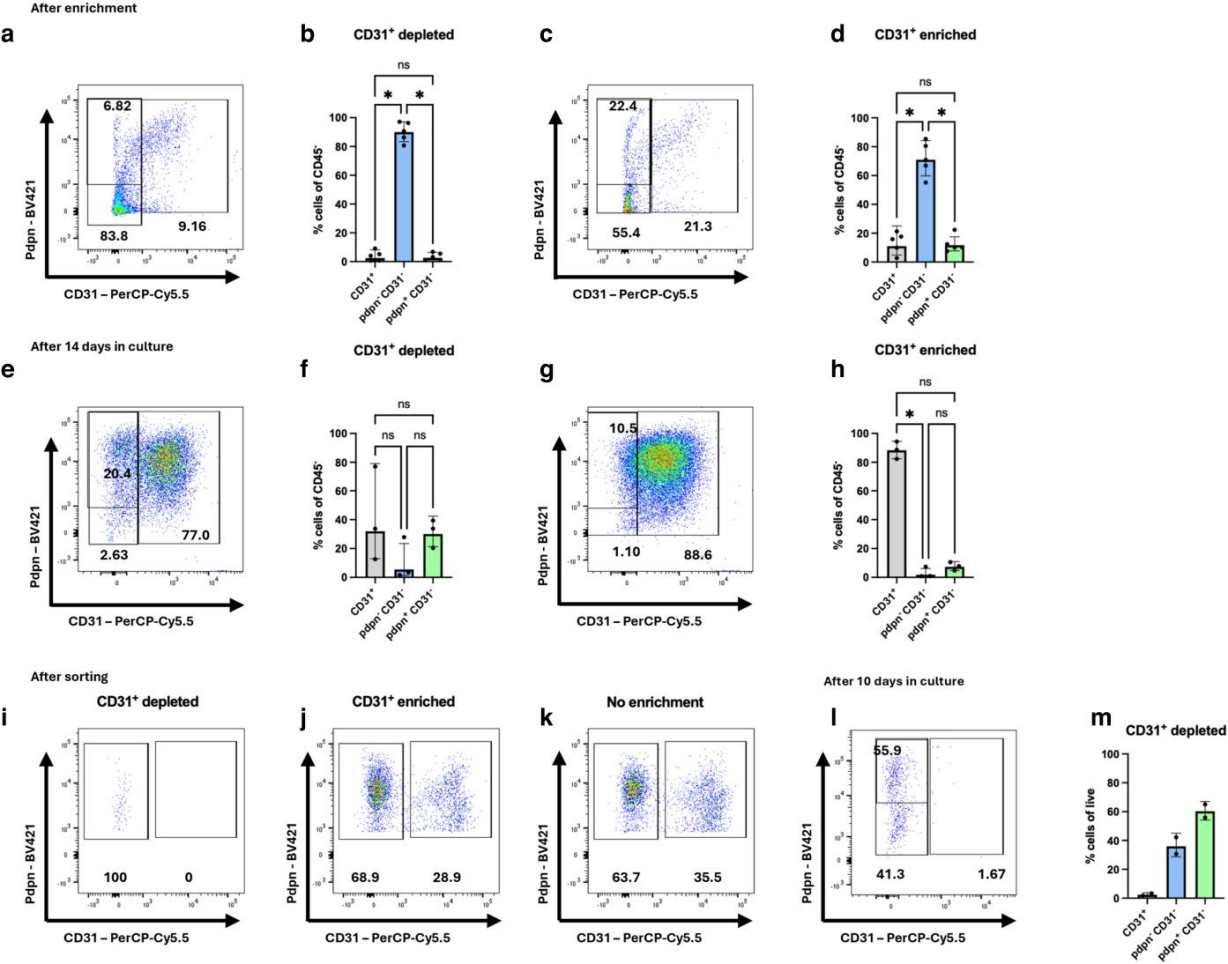
CD31-depletion does not lead to pure fibroblast cultures. (a–h) Lymphoid stromal cells were obtained from peripheral lymph nodes, cultured for 5 days prior to enrichment based on CD31-binding. Directly after enrichment and 14 days after culture, the LEC and fibroblast composition were determined by flow cytometry. (a–d) Staining of cultures directly after CD31-depletion, showing CD31-depleted (a, b) and CD31-enriched (c, d) cultures. (e–h) Staining after 2 weeks in culture, showing CD31-depleted (e, f) and CD31-enriched (g, h) cultures. (i–o) Lymphoid fibroblasts were purified by sorting for CD31^−^CD45^−^Pdpn^+^ cells, then stained immediately or after 10 days in culture. (h, i) Staining directly after sorting, showing CD31-depletion (i), CD31 enrichment (j), and CD45-depletion only (k), prior to culture. Representative flow profile (l) and proportion (m) of CD31^+^, CD31^−^, and Pdpn^+^ CD31^−^ cells of CD45^−^ CD31^−^ cells after culture. Each data point represents an individual mouse, and each data point is considered independent. Each symbol represents a biological replicate, and lines indicate the geometric mean. Statistical significance was determined using a Kruskal–Wallis test with Dunn’s multiple comparison test (b, d, f, h, m). *P < 0.05.

Given the propensity for CD31^+^ cells to dominate cell cultures despite enrichment of PDGFRα^+^ fibroblasts, we next opted to deplete CD31^+^ cells to remove ECs after 5 days in culture, when most of the cells are CD45^−^. This yielded >80% of the culture comprising CD31^−^ stromal cells, although there was a very low frequency of Pdpn^+^ lymphoid stromal cells (Fig. 3a and b). Surprisingly, the flow-through data containing CD31-bound cells gave similar results to the CD31 enrichment, with more than half of the enriched population comprising CD31^−^Pdpn^−^ cells (Fig. 3c and d). This suggests that the enrichment protocols are not sufficient for enriching specific cell types. Indeed, cell cultures were highly variable; the most consistent finding was that cell suspensions depleted of CD31^+^ cells struggled to survive >10 days in culture. The CD31^+^ cell-depleted cultures that survived usually presented similar proportion of CD31^+^ cells as before CD31 depletion, with evidence of Pdpn^+^ fibroblasts being maintained (Fig. 3e and f). In comparison, the CD31^+^-enriched cultures were essentially pure CD31^+^Pdpn^+^ cultures after 10 days (Fig. 3g and h).

To generate a pure culture of lymphoid fibroblasts, we next used flow cytometric cell sorting to purify Pdpn^+^ CD31^−^ cells. Immediately post sorting, cells were >95% CD31^−^ (Fig. 3i), compared to CD31^+^-enriched (Fig. 3j) and just CD45^+^-depleted (Fig. 3k) samples. When cultured, high purity CD31^−^ cell cultures were maintained, which included both fibroblasts (Pdpn^+^ CD31^−^) and pericytes (Pdpn^−^ CD31^−^) (Fig. 3l and m). While this method yielded pure lymphoid fibroblasts, the sorted CD31^−^ cells struggled to expand *in vitro*, unlike the CD31^+^-enriched samples, which thrived in culture, rendering this method inefficient for downstream analysis of fibroblast function. We conclude that optimal lymphoid fibroblast growth *in vitro* is supported by EC-derived factors, as in our hands we were not able to consistently culture pure populations of fibroblasts.

### Lung stromal cells are more stable than lymphoid stromal cells *in vitro*

**Figure 4:**
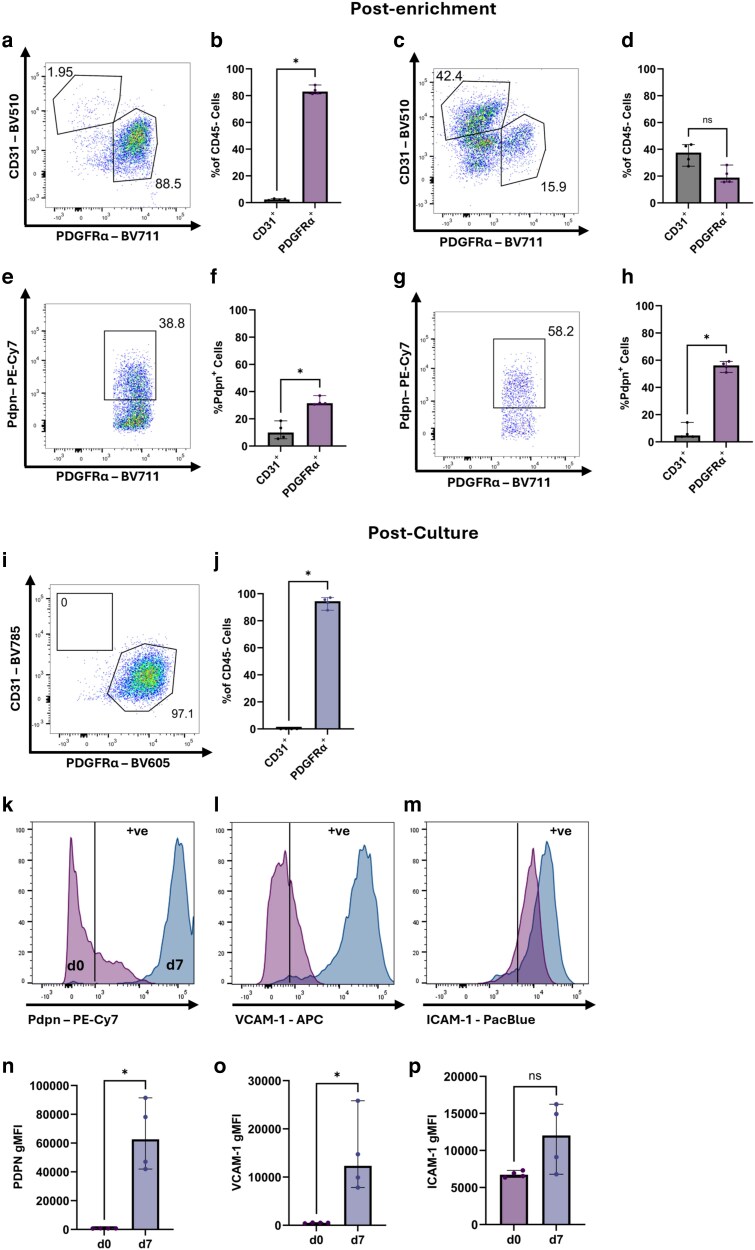
Pure primary pulmonary fibroblast cultures can be obtained with PDGFRα enrichment. Mouse lungs were digested to extract whole cells, enriched for PDGFRα^+^ cells, and both the PDGFRα-depleted and -enriched fractions were cultured. (a–d) Staining directly after enrichment, showing PDGFRα-enriched (a, b) and -depleted (c, d) samples. (e–h) Pdpn staining on PDGFRα-enriched (e, f) and -depleted fractions (g, h), directly after enrichment. (i–p) Characteristics of cultured lung fibroblasts. (i, j) Purity of PDGFRα-enriched samples after 7 days of culture. Expression of Pdpn (k, n), VCAM-1 (l, o), and ICAM-1 (m, p) on lung fibroblasts at Day 0 and Day 7 of cultures. Vertical lines in (k–m) designate the positive gate. Each symbol in (b, d, f, h, j, n, o, p) represents a biological replicate, and lines indicate the geometric mean. Statistical significance was determined using a Mann–Whitney test (b, d, f, h, j, n, o, p). *P < 0.05.

We next set out to determine whether we could apply these culture principles to other tissue fibroblasts, such as the lung, which is rich in fibroblasts. Lung fibroblasts were isolated using a slightly modified digestion protocol and enriched for PDGFRα^+^ cells prior to *in vitro* culture. PDGFRα^+^-enriched and -depleted samples were stained for flow cytometry for phenotypic analysis. In this instance we elected to use PDGFRα staining to identify fibroblasts, as pulmonary fibroblasts are not uniformly positive for Pdpn [22], unlike lymphoid fibroblasts. In contrast to our results obtained for lymph nodes, PDGFRα^+^ enrichment resulted in highly pure lung fibroblasts, with >80% of cells being PDGFRα^+^ CD31^−^ immediately after digestion and enrichment (Fig. 4a and b). While some PDGFRα^+^ fibroblasts were observed in the PDGFRα^+^-depleted fraction (Fig. 4c and d), this was not a substantial loss of cells. Pdpn expression was only observed in the CD31^−^ PDGFRα^+^ population, suggesting Pdpn^+^ LECs were poorly isolated from the lung (Fig. 4e–h). It is possible that enrichment led to depletion of Pdpn^+^ fibroblasts, given the lower proportion of Pdpn^+^ cells observed: 40% post enrichment vs. 60% on freshly isolated cells (Fig. 4e–h).

After 7 days of culture, the fibroblast cultures remained highly pure, with >80% PDGFRα^+^ cells (Fig. 4i, j). Cultured lung fibroblasts upregulated expression of VCAM-1 (Fig. 4l, o) and Pdpn (Fig. 4k, n) after 1 week in culture, while ICAM-1 (Fig. 4m, p) was maintained at high levels from isolation and during cell culture. In summary, pure primary lung fibroblast cultures can be obtained with a simple enrichment procedure and maintained in culture in the absence of EC support, unlike lymphoid fibroblasts, although culture can affect their cell surface phenotype.

### Stromal cells respond to TLR4 stimulation *in vitro*

**Figure 5:**
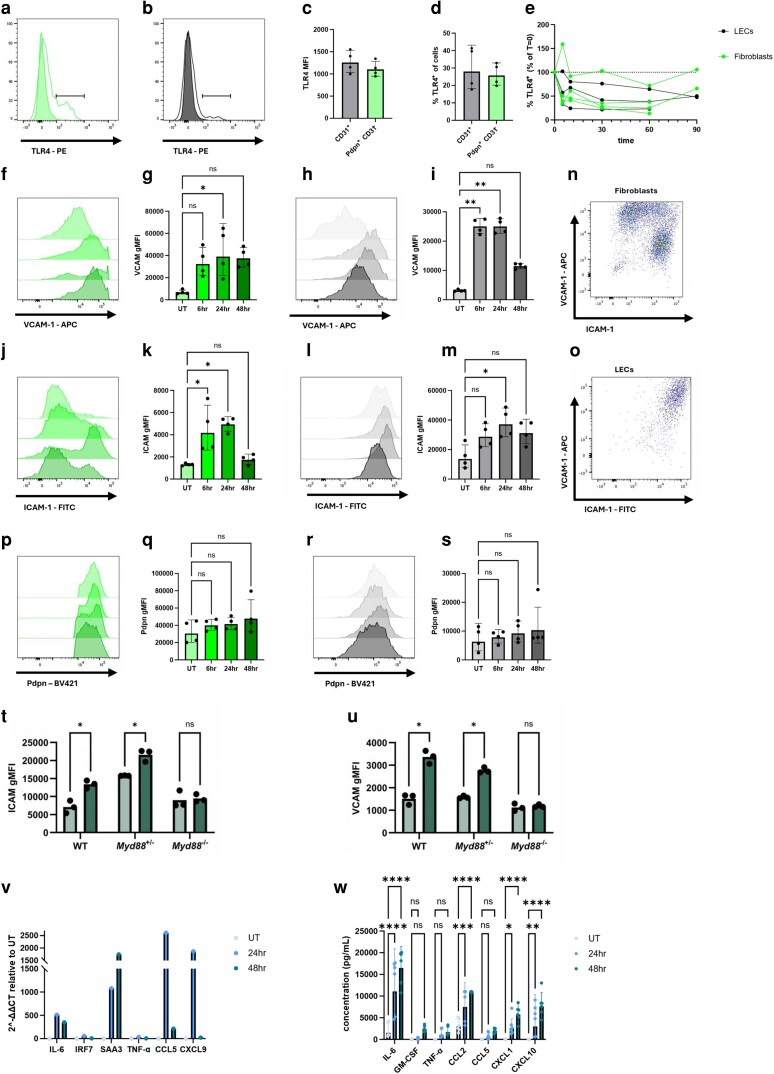
Lymphoid stromal cells express TLR4 and respond to LPS stimulation. Peripheral lymphoid stromal cells were extracted from WT, *Tlr4*^−/−^, and/or *Myd88*^−/−^ mice and cultured *in vitro* for 10–14 days. (a–e) TLR4 expression and responses by primary lymphoid stromal cells. (a, b) TLR4 expression was determined on WT (not filled) and *Tlr4*^−/−^ (filled) fibroblasts (a) and LECs (b). The level of expression measured by gMFI (c) and proportion of TLR4^+^ cells (d) were determined for fibroblasts (green) and LECs (black). (e) Lymphoid stromal cells were expanded *in vitro* for 2 weeks, stimulated with 1 μg/ml LPS and TLR4 surface expression was determined at 5, 10, 30, 60, and 90 min by flow cytometry (*n* = 3–4, combined of two independent experiments). (f–s) Primary lymphoid fibroblast phenotype was measured by gMFI of antibody staining after 6, 24, or 48 h after 1 μg/ml LPS stimulation. (f–i) VCAM-1 expression on fibroblasts (f, g) and LECs (h, i). (j–m) ICAM-1 expression on fibroblasts (j, k) and LECs (l, m). (n, o) Representative flow plots show VCAM-1 and ICAM-1 staining of fibroblasts (n) and LECs (o) after 24 h of LPS stimulation. (p–s) Pdpn expression on fibroblasts (p, q) and LECs (r, s). (t, u) Expression of ICAM-1 (t) and VCAM-1 (u) 24 h after LPS stimulation of WT, *Myd88*^−/+^, and *Myd88*^−/−^ lymphoid fibroblasts, light green = unstimulated, dark green = stimulated. (v) Gene expression in mixed lymphoid stromal cell cultures was determined by qRT-PCR 24 and 48 h after treatment with 1 μg/ml LPS. (w) Protein concentration in culture supernatants from mixed lymphoid stromal cell cultures was determined by LEGENDplex assay using the mouse anti-virus response panel 24 and 48 h after treatment with 1 μg/ml LPS. Each data point represents a culture from an individual mouse, and each data point is considered independent. Each symbol represents a biological replicate, and lines indicate the geometric mean. Statistical significance was determined using a Kruskal–Wallis test with Dunn’s multiple comparison test (c, d, g, i, k, m, q, s, t, u, v, w). *P < 0.05, **P < 0.01, ***P < 0.001, and ****P < 0.0001.

Multiple transcriptomic analyses have demonstrated that lymphoid fibroblasts express TLRs and the signalling machinery, with a similar expression level as monocytes (www.immgen.org). We first confirmed the cultured lymphoid stromal cells express TLR4 (Fig. 5a and b), as we had previously shown for freshly isolated lymphoid fibroblasts [11]. Lymphoid fibroblasts and LECs cultured for 10 days expressed TLR4 above background, defined by TLR4 antibody staining on lymphoid stromal cells expanded from *Tlr4*^−/−^ mice (Fig. 5a and b). Fibroblasts and LECs expressed similar levels of TLR4 (Fig. 5c) and had similar proportion of TLR4^+^ cells (Fig. 5d). To demonstrate TLR4 responsiveness we measured TLR4 internalization by loss of surface TLR4 staining, as has been shown for monocytes and mouse embryonic fibroblasts [23]. Cultured lymphoid stromal cells were stimulated with LPS and cells harvested 5–90 min post stimulation and the level of surface TLR4 quantified by the proportion of TLR4^+^ cells as a percentage of unstimulated cells. Both LECs and fibroblasts responded to LPS stimulation, with peak loss of surface TLR4 staining within 10 min for both LECs and fibroblasts (Fig. 5e). Internalization was, however, variable, with not all cultured stromal cells producing the internalization response. The direct response to TLR4 stimulation is, however, in line with a recent publication that showed direct signalling by phospho-flow staining of freshly isolated lymphoid stromal cells [24].

To understand how TLR4 signalling influences lymphoid stromal cell phenotype, WT lymphoid stromal cell cultures were stimulated with LPS. Both supernatant and cells were harvested 6, 24, and 48 h later. Cells were analysed by flow cytometry, measuring surface expression of the adhesion molecules VCAM-1 and ICAM-1 (Fig. 5f–m). Fibroblasts significantly upregulated VCAM-1 within 24 h and maintained higher expression for at least 48 h (Fig. 5f and g). LECs also increased VCAM-1 surface expression, peaking within 6 h of LPS exposure; the peak was maintained for until 24 h and reduced at 48 h (Fig. 5h and i). Cultured lymphoid fibroblasts were initially ICAM-1-negative, but began to upregulate this marker at 6 h, reaching a peak of expression at 24 h before returning to baseline at 48 h (Fig. 5j and k). LECs were initially ICAM-1^+^ and showed a significant increase in ICAM-1 levels at 24 h, which was maintained to 48 h (Fig. 5l and m). We found a distinct difference in the upregulation of ICAM-1 and VCAM-1 between LECs and fibroblasts, where fibroblasts developed two distinct populations: one with very high VCAM-1 expression, and another characterized by high expression of both VCAM-1 and ICAM-1 (Fig. 5n). In contrast, LECs uniformly upregulated both ICAM-1 and VCAM-1 (Fig. 5o). Pdpn did not significantly change at any time point after stimulation, in either fibroblasts (Fig. 5p and q) or LECs (Fig. 5r and s). The upregulation of ICAM-1 and VCAM-1 was abrogated in *Myd88*^−/−^ lymphoid fibroblasts (Fig. 5t and u), suggesting the Myd88 pathway is key for phenotypic changes in lymphoid fibroblasts in response to LPS stimulation.

To further understand how TLR4 stimulation alters lymphoid stromal cell function, the expression of interleukin (IL)-6, interferon regulatory factor 7, serum amyloid A 3 (SAA3), tumour necrosis factor alpha (TNF-α), C-C chemokine ligand (CCL)5 and C-X-C chemokine ligand (CXCL)9, previously shown to be increased in lymphoid stromal cells [13] was determined by qRT-PCR and cytokine bead arrays 24 and 48 h after LPS stimulation. These assays were performed in mixed stromal cell cultures, containing both fibroblasts and LECs. *Il6*, *Saa3*, *Tnf*, *Ccl5*, and *Cxcl9* mRNA expression were all increased relative to unstimulated cultures (Fig. 5v). While cytokine (*Il6*, *Saa3*, and *Tnf*) mRNA expression peaked after 48 h incubation with LPS, chemokine (*Ccl5* and *Cxcl9*) mRNA had a shorter course of expression, peaking at 24 h (Fig. 5v). mRNA changes were confirmed by protein detection in culture supernatants by LEGENDplex assay, using the anti-viral panel to measure IL-6, granulocyte-macrophage colony-stimulating factor (GM-CSF), TNF-α, CCL5, CXCL1, CCL2, and CXCL10. IL-6, CCL2, CXCL1, and CXCL10 increased following LPS stimulation and peaked at 48 h (Fig. 5w), with GM-CSF, TNF-α, and CCL5 not significantly increasing after LPS stimulation. IL-6 exhibited the highest increase in concentration, followed but CCL2, CXCL10, and CXCL1 (Fig. 5w). Their upregulation 24 and 48 h after LPS stimulation is in line with published work showing that lymphoid fibroblasts produce these chemokines after intravenous LPS administration [13]. Altogether, these data show that lymphoid stromal cells react to TLR4 ligation by upregulating adhesion molecules such as VCAM-1 and ICAM-1 and producing chemokines and cytokines that may impact on immune cell recruitment and function.

### Stromal cells respond to TLR3 stimulation *in vitro*

**Figure 6:**
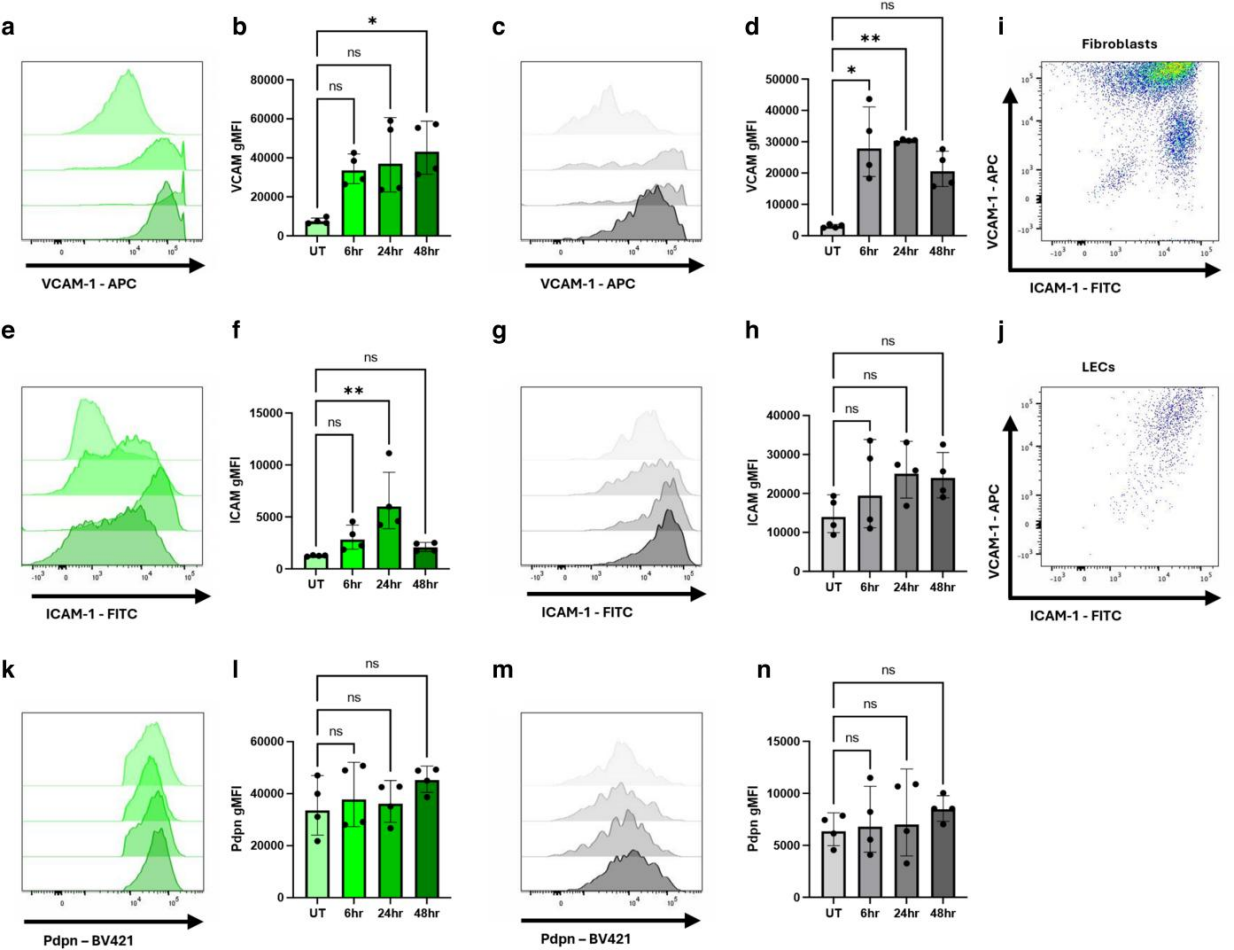
Cultured lymphoid stromal cells respond to polyI:C stimulation. Lymphoid stromal cells were extracted from WT peripheral lymph nodes, cultured *in vitro* for 2 weeks and stimulated with 2 μg/ml Poly (I:C) for 6, 24, and 48 h. Cell surface phenotype was determined by measuring the gMFI of antibody staining for VCAM-1 on fibroblasts (a, b) and LECs (c, d), ICAM-1 on fibroblasts (e, f) and LECs (g, h). Representative flow plots show VCAM-1 and ICAM-1 staining of fibroblasts (i) and LECs (j) after 24 h of polyI:C stimulation. Pdpn expression was determined by gMFI of antibody staining on fibroblasts (k, l) and LECs (m, n). Each data point represents a culture from an individual mouse, and each data point is considered independent. Each symbol represents a biological replicate, and lines indicate the geometric mean. Statistical significance was determined using a Kruskal–Wallis test with Dunn’s multiple comparison test (b, d, f, h, l, n). *P < 0.05 and **P < 0.01.

Lymphoid stromal cells also express TLR3 [24–27] with LECs and ECs expressing slightly higher levels than fibroblasts (www.immgen.org). To determine whether lymphoid stromal cells directly respond to this TLR *in vitro*, primary stromal cell cultures obtained from WT mice lymph nodes were stimulated with Polyinosinic:polycytidylic acid (poly I:C) for 6, 24, and 48 h, and the expression of VCAM-1, ICAM-1, and Pdpn determined by flow cytometry. Lymphoid fibroblasts significantly upregulated VCAM-1 48 h after polyinosinic:polycytidylic acid (polyI:C) stimulation (Fig. 6a and b), with increased expression evident within 6 h and a significant increase observed after 48 h. LECs upregulated VCAM-1 at 24 h, with increased expression evident within 6 h (Fig. 6c and d). ICAM-1 surface expression in fibroblasts was also significantly increased 24 h after incubation with polyI:C (Fig. 6e and f), mimicking results observed following TLR4 stimulation, while LECs did not significantly change their already high level of ICAM-1 expression after TLR3 stimulation (Fig. 6g and h). We analysed co-expression of VCAM-1 and ICAM-1 in lymphoid fibroblasts (Fig. 6i) and LECs (Fig. 6j) following 24-h stimulation with polyI:C, to determine whether increased expression of one molecule was associated with upregulation of the other. As observed with LPS stimulation, lymphoid fibroblasts developed two distinct populations: one exhibiting co-upregulation of VCAM-1 and ICAM-1, and another characterized by lower expression of VCAM-1 and high expression of ICAM-1 (Fig. 6j). LECs behaved similarly to polyI:C as to LPS stimulation: ICAM-1 and VCAM-1 were typically both highly expressed (Fig. 6j). Pdpn expression did not increase in fibroblasts or in LECs stimulated with TLR3 ligands (Fig. 6k–n). Taken together, these data demonstrate that lymphoid stromal cells sense inflammatory stimuli via TLR3 and TLR4 and can respond by upregulating adhesion molecules that may impact on immune cell behaviour.

## Discussion

In this study we set out to establish methods to culture primary tissue fibroblasts to test their ability to respond to TLR ligands directly. We established that lymph node-derived fibroblasts fared poorly in culture in the absence of endothelial cells, suggesting crosstalk between these cells *ex vivo*. In contrast, lung-derived fibroblasts were much more stable as a pure culture *in vitro*. Using a mixed culture of lymph node-derived fibroblasts and LECs, we established that both LECs and fibroblasts can respond to TLR ligands, upregulating cell adhesion molecules, but not Pdpn, via the Myd88 pathway. Lymphoid stromal cells also upregulated expression of immune modulatory inflammatory proteins, suggesting they can impact immune cell behaviour by responding to pathogen-derived signals.

We expected that establishment of a pure culture of lymphoid fibroblasts would be straightforward. Addition of ITS supplement was essential to facilitate the growth of lymphoid stromal cells. While we were able to generate a pure fibroblast culture by cell sorting, these cultures did not expand sufficiently to allow us to perform further experiments, leading us to work with mixed cultures throughout this study. Why lymphoid fibroblasts are such poor adapters to tissue culture compared to lung fibroblasts remains unclear. Lung and lymphoid fibroblasts have different origins, *in vivo* tissue environments, and exposures to other cell types, which together may impact their function [28]. Lymphoid fibroblasts were generally more stable in co-culture with LECs, perhaps due to cross-regulation between these cells. Human umbilical vein endothelial cells support bone marrow fibroblast growth *in vitro* [29], and while LECs are not potent producers of fibroblast growth factors (immgen.org), they can produce significant levels of *Pdgfb*; PDGFs are potent stimulators of fibroblast growth [30]. Culturing lymphoid fibroblasts on collagen-coated tissue culture plates may improve lymphoid fibroblast cultures, as described for human lymphoid fibroblasts [21, 30–32]. Our results are in line with other publications using *in vitro* primary lymphoid fibroblasts, where poor viability and outgrowth in the absence of endothelial or other stromal subsets have been described, and fibroblast/LEC mixed cultures are more stable [16, 21].

We establish here that primary lymphoid fibroblasts and LECs can respond to TLR4 and TLR3 signalling *in vitro*. Intriguingly, lymphoid LECs and fibroblasts gave distinctly different responses to TLR stimulation when we considered co-expression of ICAM-1 and VCAM-1. LECs gave uniform responses, where ICAM-1 and VCAM-1 positively correlated with each other. In comparison, lymphoid fibroblasts were divided into two populations, expressing higher ICAM-1 or higher VCAM-1 following TLR stimulation. This differs from their *in vivo* responses, where lymphoid fibroblasts typically upregulate ICAM-1 and VCAM-1 together [11]. The impact of TLR signalling on stromal cell responses has been suggested previously, with Malhotra *et al*. [13] demonstrating that lymphoid stromal cells undergo transcriptional changes within 18 h of LPS injection. The induction of pro-inflammatory cytokines like IL-6 and CCL2 suggests that lymphoid stromal cell responses to DAMPS and PAMPs may impact immune cell behaviour. Expression of IL-6 by lymphoid fibroblasts promotes the generation of the germinal centre via induction of follicular helper T cells [33]. Expression of CCL2 by lymphoid fibroblasts leads to recruitment of monocytes that negatively impact immune responses, disrupting responses to immune checkpoint blockade, a response downstream of tumour-derived TLR4 ligands in metastatic disease [34], and has also been shown to impair plasma cell differentiation via reactive oxygen species [35]. Evidence of a functional role of direct TLR sensing by lymphoid stromal cell has recently been demonstrated, where footpad administration of LPS promoted expression of the homeostatic chemokines CCL19/21, increasing recruitment of lymphocytes to the draining lymph code and the magnitude of the CD8^+^ T cell response, impacting anti-tumour immunity [24]. Our data complements previously published data demonstrating the essential role lymphoid fibroblasts play in maintaining and regulating the macrophage niche in the lymph node [36]. Stromal cells also expressed CXCL9/10 following LPS stimulation, suggesting a role for regulating T cell positioning after infection via TRIF-mediated signalling. These chemokines drive C-X-C chemokine receptor 3^+^ type 1 helper CD4^+^ and CD8^+^ T cell recruitment [32, 33] and their positioning in the lymphoid tissue [34–36].

To conclude, this paper builds on the previous data that shows lymphoid stromal cells support immune cells through the provision of migration, growth, and differentiation cues that are key for the regulation of protective immunity. We provide evidence that lymphoid fibroblasts and LECs can respond to TLR ligands directly by altering their expression of cell adhesion molecules and expressing immune-modulating cytokines and chemokines. This suggests that targeting of lymphoid stromal cells via PRRs may be tractable approach to modulating leucocyte behaviour.

## Limitations of the study

There are a number of further questions raised by the work contained herein that are beyond the scope of this study. Why lymphoid fibroblasts are so refractive to tissue culture adaptation, even in the presence of co-culture of LECs, but lung fibroblasts readily adapt is unclear. Moreover, our data as presented is somewhat incomplete, as we have not assessed the production of lymphoid cytokines and chemokines in response to TLR3 stimulation. We note that others have shown that lymphoid stromal cells produce inflammatory cytokines and chemokines in response to TLR3 stimulation [26, 27]. Further study of how production of inflammatory cytokine and chemokines by lymphoid fibroblasts impacts on the T and B cell response in different immune contexts, e.g. infection, autoimmunity, and cancer, will bring further insight into how fibroblasts coordinate activated immune responses.

## Data Availability

All data supporting the figures in this manuscript are provided, or will be made available upon reasonable request.

## Abbreviations:

CCL: C-C chemokine ligand
CXCL: C-X-C chemokine ligand
gMFI: geometric mean fluorescence intensity
GM-CSF: granulocyte-macrophage colony stimulating factor
ICAM: intercellular adhesion molecule
IL: interleukin
LEC: lymphatic endothelial cell
LPS: lipopolysaccharide
PDGFRα: platelet-derived growth factor receptor alpha
Pdpn: podplanin
polyI:C: polyinosinic:polycytidylic acid
SAA3: serum amyloid A 3
TLR: Toll-like receptor
TNF-α: tumour necrosis factor alpha
VCAM: vascular cell adhesion molecule
